# Mitochondrial Genomes, Phylogenetic Associations, and SNP Recovery for the Key Invasive Ponto-Caspian Amphipods in Europe

**DOI:** 10.3390/ijms221910300

**Published:** 2021-09-24

**Authors:** Tomasz Mamos, Michał Grabowski, Tomasz Rewicz, Jamie Bojko, Dominik Strapagiel, Artur Burzyński

**Affiliations:** 1 Department of Invertebrate Zoology and Hydrobiology, Faculty of Biology & Environmental Protection, University of Lodz, 90-237 Łódź, Poland; michal.grabowski@biol.uni.lodz.pl (M.G.); tomasz.rewicz@biol.uni.lodz.pl (T.R.); 2 Zoological Institute, University of Basel, 4051 Basel, Switzerland; 3 Centre for Biodiversity Genomics, University of Guelph, Guelph, ON N1G 2W1, Canada; 4 National Horizons Centre, Teesside University, Darlington DL1 1HG, UK; j.bojko@tees.ac.uk; 5 Biobank Lab, Department of Molecular Biophysics, Faculty of Biology & Environmental Protection, University of Lodz, 90-237 Łódź, Poland; dominik.strapagiel@biol.uni.lodz.pl; 6 Department of Genetics and Marine Biotechnology, Polish Academy of Sciences, Institute of Oceanology, Powstańców Warszawy 55, 81-712 Sopot, Poland; aburzynski@iopan.pl

**Keywords:** Amphipoda, invasive species, population genetics, mitogenome, Ponto-Caspian, SNP

## Abstract

The Ponto-Caspian region is the main donor of invasive amphipods to freshwater ecosystems, with at least 13 species successfully established in European inland waters. *Dikerogammarus* spp. and *Pontogammarus robustoides* are among the most successful, due to their strong invasive impact on local biota. However, genomic knowledge about these invaders is scarce, while phylogeography and population genetics have been based on short fragments of mitochondrial markers or nuclear microsatellites. In this study, we provide: (i) a reconstruction of six mitogenomes for four invasive gammarids (*D. villosus*, *D. haemobaphes*, *D. bispinosus*, and *P. robustoides*); (ii) a comparison between the structure of the newly obtained mitogenomes and those from the literature; (iii) SNP calling rates for individual *D. villosus* and *D. haemobaphes* from different invasion sites across Europe; and (iv) the first time-calibrated full mitogenome phylogeny reconstruction of several Ponto-Caspian taxa. We found that, in comparison to other gammarids, the mitogenomes of Ponto-Caspian species show a translocation between the tRNA-E and tRNA-R positions. Phylogenetic reconstruction using the mitogenomes identified that Ponto-Caspian gammarids form a well-supported group that originated in the Miocene. Our study supports paraphyly in the family Gammaridae. These provided mitogenomes will serve as vital genetic resources for the development of new markers for PCR-based identification methods and demographic studies.

## 1. Introduction

The Ponto-Caspian region (Azov, Black, and Caspian seas with surrounding areas) is a significant donor of invasive amphipods to European inland water bodies [1,2,3]. At least 13 morphospecies have successfully established populations to date [4,5]. Among them, *Dikerogammarus villosus* and *Dikerogammarus haemobaphes* have colonized most of the European main inland water bodies in less than 20 years, having a deteriorating effect upon local benthic communities [6,7,8]. *Dikerogammarus bispinosus* (a third invasive representative of the genus *Dikerogammarus*) reached the Rhine estuary via the southern invasion corridor [1]; however, populations of this species have been declining in the lower section of the Danube in recent decades [9,10]. *Dikerogammarus villosus* and *D. haemobaphes* earned their nicknames “killer” and “demon” shrimp, respectively, due to their impacts on local fauna. They are the highly adaptable, physiologically tolerant, and efficient predators characterized by a high fecundity, which allows them to dominate local macroinvertebrate communities [8,11,12,13], as well as introduce pathogenic species [14,15,16,17,18,19]. In particular, *D. villosus* is regarded as one of the worst 100 invasive species in Europe [20,21] and has been deemed the worst non-native amphipod invader of English and Welsh waterways by the UK Environment Agency [22]. In recent years these species have accelerated their invasion, as new records, especially of *D. villosus*, were noted in English and Welsh waterways [22], Baltic States [7], and the Masurian Lake district in Poland [23]. *Dikerogammarus haemobaphes* has most recently invaded Boroughbridge in North Yorkshire, UK, carrying with it several invasive parasites [24]. The European invasion of *D. villosus* is followed by *D. haemobaphes* [6] and, most recently, by *Pontogammarus* *robustoides* [25,26].

Phylogeographic and population genetics data have been attained for *D. villosus* throughout its invaded range [27,28,29]. Two invasive populations, one originating from the Danube and the other originating from the Dnieper, were found in Central and Western Europe. They are genetically differentiated and allopatrically distributed, and neither of them show signs of a loss of genetic diversity compared to respective source areas. A recent phylogeographic study by [6] on *D. haemobaphes* revealed the presence of cryptic lineages in the native region, as well as shallow differentiation in populations from the European invaded area. The evolutionary processes behind the invasion of these species are still under examination [6,27,28,30,31]. Little is known about the most recent colonization of Europe by *P. robustoides*, including its molecular diversity [25]. It’s dispersal routes and molecular divergence require exploration, since this species has the potential to be invasive [11,32].

The original descriptions of *D. villosus* (Sowinsky, 1894), *D. haemobaphes* (Eichwald, 1841), and *P. robustoides* (Sars, 1894) placed them in the genus *Gammarus* (respectively, *Gammarus marinus* var. *villosa* Sowinsky, 1894, *Gammarus haemobaphes* Eichwald, 1841, and *Gammarus robustoides* G.O. Sars, 1894) in the family Gammaridae Leach, 1814. Then, ref. [33] established the genus *Dikerogammarus*, to which he moved *D. villosus* and *D. haemobaphes*, joined later by *D. bispinosus* Martynov, 1925. In 1904, ref. [34] established the genus *Pontogammarus* into which *P. robustoides* was placed. Moreover, ref. [35], based on several morphological features shared by Ponto-Caspian species, challenged the former genus-level classification within Gammaridae, and placed *Dikerogammarus* together with several newly established genera into an informal taxonomic group he called “*Dikerogammarus*-*Pontogammarus* complex” or “Ponto-Caspian complex of genera”. Following this, ref. [36], after re-evaluation of the morphological traits within the group, moved the whole “*Dikerogammarus*-*Pontogammarus* complex” to the family Pontogammaridae Bousfield in 1977.

Several decades later, ref. [37] conducted a general taxonomic revision of Amphipoda based on a cladistic analysis of a number of morphological traits, which excluded *Dikerogammarus* from the Pontogammaridae and placed it back into the Gammaridae. More recently, molecular studies by [38,39] suggested high phylogenetic affinity of *Dikerogammarus* to other genera of the former “*Dikerogammarus-Pontogammarus* complex” (sensu [35]) that are classified within Pontogammaridae. Nevertheless, given the limited number of molecular markers used and the lack of resolution in the phylogenetic tree, the exact relationships within as well as between the pontogammarid clade and other gammarids were not fully identified. The abovementioned authors continued to refer to them as “Ponto-Caspian gammarids” or “Ponto-Caspian group of genera”, without a conclusion into which family they should belong. Further, the within-genus taxonomy of *Dikerogammarus* continues to be a subject of discussion and is far from being fully resolved, mainly due to poor descriptions of some species, as well as missing type materials. For example, until recently, *D. bispinosus* was considered a subspecies of or synonym for *D. villosus* Sowinsky 1894, while *D. villosus* was considered as a synonym for *D. haemobaphes* Eichwald 1841 [40]. A molecular study by [31] resolved this question and confirmed the taxonomic status of *D. villosus*, *D. haemobaphes*, and *D. bispinosus* as three separate species. Nevertheless, the most recent studies show that, while *D. villosus* seems to be a well-defined species over its geographic range [27], both *D. haemobaphes* and *D. bispinosus* contain divergent phylogenetic lineages, which may represent yet undescribed, cryptic, or pseudocryptic species [6,41].

There is still relatively scarce knowledge on the genomics of the Amphipoda. To date, five amphipod (complete or partial) genomes are available for the species: *Orchestia grillus*, *Trinorchestia longiramus*, *Gammarus roeselii*, *Hyalella azteca*, and *Parhyale hawaiensis* [42]. The main obstacle to progression is their large and repetitive genome. Thus far, only two such genomes have been completed, first for *Parhyale hawaiensis* with a size reaching 3.6 Gb [43] and second for *Gammarus roeselii* with a size estimated at 3.4 Gb [44], both being among the largest reported arthropod genomes [43]. Another potential problem could be polyploidization, which was documented for the Ohrid Lake *Gammarus* species flock [45]. Genetic data have also been gathered via a transcriptomic approach, providing nuclear and mitochondrial data for ~40 species of amphipod [46,47,48,49].

In the absence of genomic data, mitochondrial genomes are much easier to sequence and reconstruct and are often used in phylogenetic models [50,51,52]. The structure of the amphipod mitochondrial genome resembles the general structure of other animal mitogenomes; being small (14–18 kb) circular molecules and having ~37 genes: 13 protein coding, 2 rRNA, and 22 tRNA [53]. Due to their small size and relative abundance, the complete mitogenome sequences are relatively easy to obtain from next generation sequencing projects [43,54,55,56,57], as well as for the widely distributed freshwater Palearctic superfamily, Gammaroidea [50,51,52,58]. It is common for an important resource, mitochondrial transcripts, to not be assembled and published, despite the fact that a large number of reads in each data set belong to the mitochondrial genome [59].

Pertinent to the *Dikerogammarus* spp., ref. [55] published the first mitochondrial genome for a single *D. haemobaphes* from an invasive population in England, which became the first reported full mitogenome for the family Pontogammaridae. Moreover, ref. [55] reported a potential recombination and duplication of a tRNA, as well as a putative duplication of an ATP8-like gene within the control region (CR). The primary goal of our study is to provide a comparative reconstruction of mitogenomes of four invasive gammarids of Pontio-Caspian origin (*D. villosus*, *D. haemobaphes*, *D. bispinosus*, and *P. robustoides*). Second, we will compare the structure of the obtained mitogenomes to those of the other gammarids available in public databases to search for putative rearrangements. Third, we will compare mitochondrial polymorphisms between individual *D. villosus* and *D. haemobaphes* that represent different invasive populations in Europe and, in the case of *D. villosus*, the native Ponto-Caspian population. Finally, we will provide the first time-calibrated reconstruction of the phylogenetic position of these Ponto-Caspian taxa vs. other gammaroids using mitochondrial genes.

## 2. Results

### 2.1. Molecular Species Identification

The COXI barcoding prior to sequencing supported morphological identification of the studied Ponto-Caspian species. After using BLAST on all new assemblies based on GenBank data, and precise identification through COXI and 16S genes, there was a complete agreement most of the time. In two cases, the BLAST search of the assemblies showed misidentifications with a 100% fragment identity (Table S1). The first case was *Pandorites podoceroides* (SRR3467097), which was revealed to be *Obesogammarus crassus* (the name used throughout the text). The second case was the transcriptome of *Gammarus pulex* (SRR8089725), which in fact showed the presence of two mitochondrial genomes, one of *G. fossarum* and second belonging to *G. pulex*; only the latter was used in our analyses.

### 2.2. Structure

**Figure 1:**
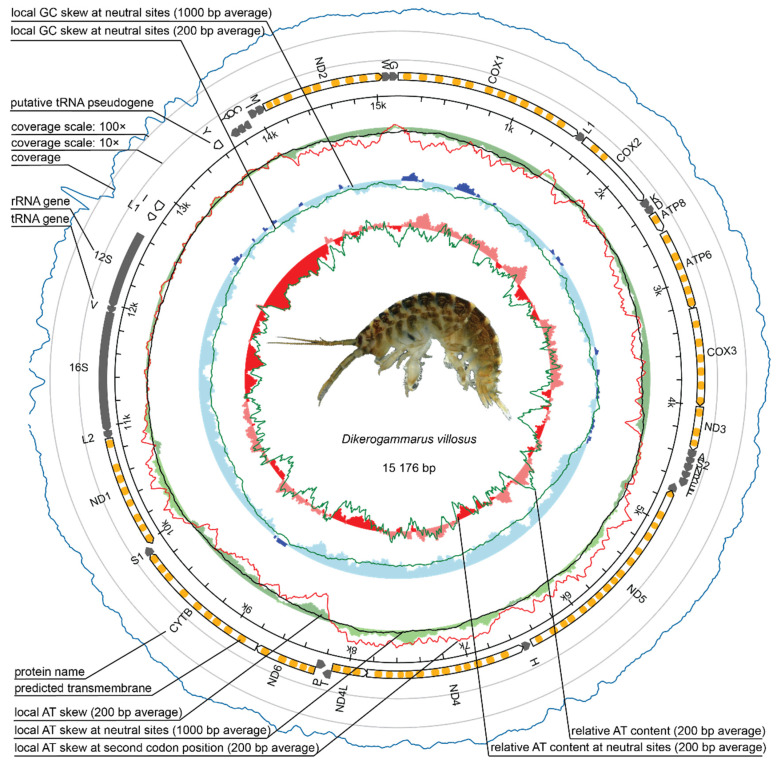
Genetic map of the mitochondrial genome for the invasive Ponto-Caspian amphipod, *Dikerogammrus villosus*. The mitogenome was generated using the mitoconstrictor set of tools. Transfer RNA genes are labelled by their single-letter amino acid code (photo. of *D. villosus* by M. Grabowski).

**Figure 2:**
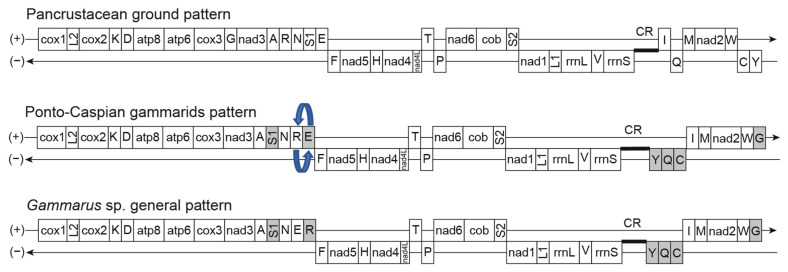
Organization of mitochondrial genomes in the Ponto-Caspian gammarids in comparison with the pancrustacean ground pattern and representative *Gammarus* genera. Gene features with altered location relative to the pancrustacean ground pattern are shown in a grey colour. Rearrangement of a tRNA is shown using blue arrows. A ‘+’ indicates the forward DNA strand and ‘−’ indicates the reverse DNA strand. Transfer RNA genes are labelled by their single-letter amino acid code. The figure follows the convention proposed in [50].

The three mitogenomes constructed for *D. villosus* consisted of 15,173 bp to 15,176 bp circular sequences in length. The mitogenome for *D. bispinosus* reached a length of 15,366 bp and the newly assembled mitogenome for *D. haemobaphes* (Germany) constituted 15,468 bp. For partial reconstruction of *P. robustoides* mitogenome, we were able to obtain a single linear contig, 14,339 bp long. The 37 expected genes (Figure 1) were annotated and resembled the canonical bilaterian gene set: 13 protein-coding genes, 2 rRNA genes, and 22 tRNA genes. Gene order and position for all Ponto-Caspian mitogenomes as well as for *Obesogammarus crassus* and *Homoeogammarus veneris* (Gammaridae), are the same (Figure 2). Relative positions of all protein and rRNA coding genes follow the Pancrustacea ground pattern, but the position and transcriptional polarity of some tRNA coding genes, in a few cases, are different (Figure 2). A single rearrangement is observed for tRNA genes relative to the pattern observed for *Gammarus* spp., i.e., the tRNA-E and tRNA-R switched places in the Ponto-Caspian amphipods (*Dikerogammarus* spp., *O. crassus*) and in *H. veneris*. Since the sequences reconstructed from transcriptomic data have inherently poor coverage for tRNA expression (Figure S1), the data for some of the Ponto-Caspian sister lineages must be interpreted cautiously.

The mitogenomes have a GC content of 32.2% for *D. villosus*; 34% for *D. haemobaphes*; 30.6% for *D. bispinous*; and 32.2% in partial *P. robustoides* mtDNA. Rich AT composition is visible in the control region (Figure 1), which is also characterized by the presence of a poly-T stretch and some tandemly repeated sequences.

### 2.3. SNP Recovery 

The comparison of reads from different ranges allowed us to identify several polymorphic sites across the mitogenome of both *D. villosus* and *D. haemobaphes* (Table S2). In total, 32 polymorphic sites differentiate *D. villosus* from Turkey and Poland, while 21 polymorphic sites differentiate between individuals from Poland and England. The *D. haemobaphes* from Germany differs from those collected in the UK by 52 polymorphic sites. All the polymorphic sites with the information on reading coverage, variant frequency, and variant *p*-value are provided in Table S2. The polymorphic sites within genes, their position, and their impact on translation are provided in Table S2. For all analysed species, the highest number of polymorphic sites is in ND5 and ND4 (4 to 7 SNPs). Equally, these are the longest genes (ND5: 1729 bp, ND4 1312 bp), except for COXI (1515–1535 bp); however, the COXI gene only has 2–3 SNP’s recorded (Table S2).

### 2.4. Phylogeny Reconstruction

**Figure 3:**
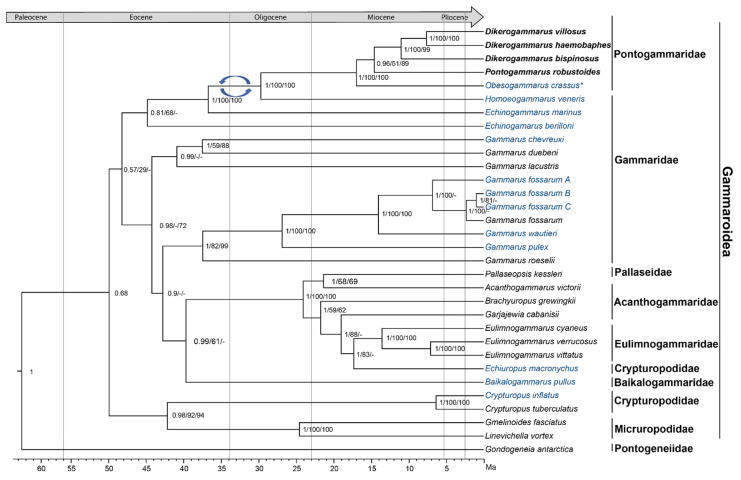
A Bayesian phylogenetic maximum clade credibility chronogram of the Gammaroidea, based on available mitogenomic and mitochondrial transcriptome data. The sources of sequences used are indicated in Table S1. Numbers next to branches indicate support: Bayesian posterior probabilities from chronogram/bootstrap values from maximum likelihood (ML) phylogeny on full data/bootstrap value for ML based on proteins (see Materials and Methods). Arrows indicate a putative tRNA rearrangement. On the right, the vertical lines indicate the families and the superfamily. Species names in bold sequences indicate assembled *de novo* from own data, names in blue indicate newly reconstructed from third party transcriptomic data. *indicates *Pandorites podoceroides* (SRR3467097), which was revealed to be *Obesogammarus crassus*.

The reconstructed phylogenetic trees share topology, showing the same well-supported clades (Figure 3, Figure S2 and Figure S3). For the maximum likelihood reconstruction, the protein-based tree gave a slightly higher support than the reconstruction based on mtDNA sequences. The Bayesian time-calibrated phylogeny reconstruction provided high posterior probabilities for most of the nodes (Figure 3 and Figure S3).

According to this phylogeny, the superfamily Gammaroidea includes a few well-supported clades that diverge in the early/middle Eocene (Figure 3 and Figure S3). First, the Baikalian families, Crypturopodidae and Micruropodidae, form a well-supported clade that has a sister relationship with a clade containing all of the other representatives of the superfamily Gammaroidea included in our analysis. The latter is composed of two clades, whose phylogenetic relationships point to paraphyly of the family Gammaridae. One includes *E. berilloni*, *E. marinus*, *H. veneris* (Gammaridae), and the Ponto-Caspian genera i.e., *Dikerogammarus* (Gammaridae), *Pontogammarus* (Pontogammaridae), and *Obesogammarus* (Pontogammaridae). This clade already started to diverge in the middle Eocene, when the *E. berilloni* lineage branched off, followed by the *E. marinus* lineage in the late Eocene. *Homoeogammarus veneris* is a sister lineage to the Ponto-Caspian genera, from which it diverged in the mid-Oligocene. The latter diverged in the middle Miocene. The three *Dikerogammarus* species diverge at the mid-to-late Miocene and form a monophyletic clade relative to *P. robustoides* and *O. crassus*.

The second clade includes all of the *Gammarus* species (Gammaridae), plus the predominantly Baikalian Pallaseidae, the endemic Baikalian Acanthogammaridae and Eulimnogammaridae, as well as the Baikalian *Echiuropus macronychus*. However, this species is classified in the Crypturopodidae and is instead affiliated with the Eulimnogammaridae in our analysis. Interestingly, *Gammarus* sp. seems to be a polyphyletic genus provided that a set of highly divergent brackish water/freshwater species (*G. chevreuxi*, *G. duebeni*, *G. lacustris*) form a sister clade to one containing freshwater *Gammarus sp*. (*G. roeselii*, *G. fossarum*, *G. wautieri*, *G. pulex*) and the Baikalian taxa. All of the above divergences may be dated to the middle and late Eocene. Finally, the *Gammarus* lineages seem to start their diversification already in the late Eocene, while the diversification of the Baikalian taxa dates back to the late Oligocene/early Miocene.

### 2.5. Substitution Rates

**Figure 4:**
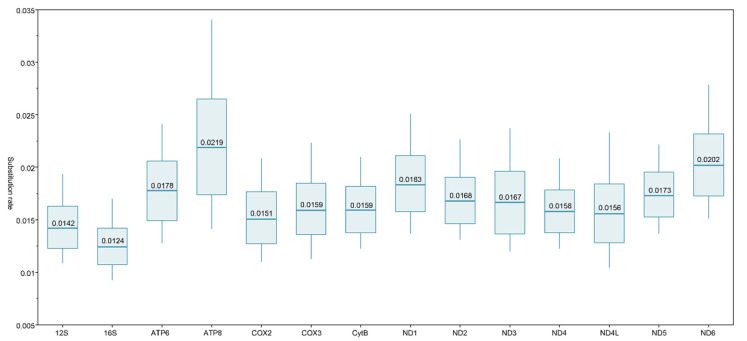
Substitution rates for 12 protein-coding and 2 rRNA mitochondrial genes. The mean value is provided within the boxplot (substitution per site × My^−1^), whiskers stand for value range, box stand for SD.

The mean relative substitution rate for the superfamily Gammaroidea falls within a range of 0.0150 to 0.0213 substitutions × My^−1^, being similar to the pre-established COXI rate of 0.01773. The substitution rate of the relatively short ATP-8 gene has a value of 0.0258 (Figure 4). All of the statistics for the protein-coding gene substitution rates are provided in Table S3.

## 3. Discussion

Gammarids of Ponto-Caspian origin are the most prominent colonizers of European inland water bodies [4]. In most cases, they are considered to be invaders, and a significant threat to native biodiversity [8,60]. The mitogenomic diversity data collected in this study provide information regarding the resolution of their taxonomic affinities [39] and presents a foundation for further genetic tool development to benefit their identification, colonization route(s), and population dynamics. The accumulation of mitogenomic data for the group is progressing [55]; however, it is important to stress that all genomic resources, prior to incorporating in analyses, should be properly validated. For superfamily Gammaroidea we have detected two cases of species misidentification.

After inspecting the assemblies based on transcriptome sequences for *G. pulex* provided by [48], it appears that the reads belong to two species, *G. pulex* and *G. fossarum*. COXI barcoding fragments allow us to identify *G. fossarum* as belonging to BIN: BOLD:ACG7784—a widely distributed species in France and Germany [61]. Both species are morphologically similar and may be hard to distinguish. The mitogenome assembled from the *G. fossarum* reads, mislabeled as *G. pulex*, was used in studies by [50,51]. The second misidentification detected in our study relates to *Pandorites podoceroides*, whose mtDNA fits with *O. crassus* from the Ponto-Caspian region; both species belong to Pontogammaridae. These mislabeled data have been used by [49]. Both mentioned misidentification, which could be avoided through simple barcoding to verify morphological determination. These findings support the need for a proper DNA barcode reference library for all organisms. Such misidentifications may result in avoidable consequences, as more countries and stakeholders include metabarcoding as a standard biomonitoring protocol [62,63,64].

In this case, *G. pulex* and *G. fossarum* have wide European ranges and are both often viewed as bioindicators [65] and model organisms for ecotoxicological studies [66]. Contrary to this, proper morphological discrimination of both species is not trivial, and we can find cryptic (possibly hyper-cryptic) diversity when using molecular studies [61,67,68]. Moreover, correct species identification is crucial when studying gene expression, especially when discrepancies of certain expression patterns may be the result of species-specific factors and not of an experimental factor or stressor.

The mitochondrial genomes of Ponto-Caspian gammarids reveal a conservative order of genes and transcriptional polarity similar to the pancrustacean ground pattern [69]. Changes in the position and location of tRNA polarity have already been shown in mitogenome reconstructions for representatives of other gammarids [50,51,58]. The outstanding feature, for the Ponto-Caspian amphipods and their sister *Homoeogammarus* in comparison to other species from the superfamily Gammaroidea, is a switch of position of two tRNA (E and R). This rearrangement was first observed for *D. haemobaphes* [55]. However, analysis and gene annotation conducted via our pipeline cannot unequivocally confirm a non-similar duplication of the ATP8, or duplication of tRNA Q genes in the structure of *D. haemobaphes* or other Ponto-Caspian gammarids [55]. It is possible that these duplications were a result of software errors, likely at the assembly or annotation step(s), which may be avoided with longer read sequencing or follow-up PCR validation in future studies. However, duplications are not rare in the amphipod mitogenome. A duplication of the control region in some Gammaridae was putatively identified in the Baikalian *Garjajewia cabanisii* [52], and also reported for the mitogenome of *G. roeselii* [50]. The mitogenomes of the Ponto-Caspian species that we have sequenced do not show such structures. However, in our case, the coverage of the control region is lower than in the study of [50], and is rather problematic for assembly due to high AT content and putative repetitions.

To date, studies using amphipod mitochondrial DNA focus primarily on phylogeographic and population dynamics, including only fragments of COXI and 16S genes [6,70]. In our study, we detected multiple SNPs across most of the mitochondrial genes for different populations, both for *D. villosus* and *D. haemobaphes*. Detection of SNPs at the species level can be valuable to the study of invasive organisms, helping one to identify their source population(s) and determine their invasion corridor(s) [71,72,73]. Growing accessibility, together with falling prices of high-throughput sequencing, will undoubtedly make multigene SNP data a primary resource to study biological invasions [74]. This can be achieved using RNA-Seq data, which are particularly rich in mitochondrial transcripts.

Our phylogenetic analysis reveals well-supported relationships between four members of the Pontogammaridae using mitogenomics. The results show that the Pontogammaridae includes a supported monophyletic clade with close affinities to *Homeogammarus*, which supports the results of [38]. Our observation that *Echinogammarus* is a sister genus to the aforementioned taxa is also supported by nuclear data from other studies [39]. As such, ref. [38] suggest moving *E. marinus* to the genus *Marinogammarus*, originally created for that species by [75]. Given that *E. marinus* is the type species for *Marinogammarus*, our results support such a claim. The type species of *Echinogammarus* is *E. berilloni*, present in our analysis and clearly belonging to another lineage. The monophyletic clade composed of Pontogammaridae and *Echinogammarus* (Gammaridae) was also shown by [50] using a concatenated dataset of mitochondrial coding genes; however, in their study, this group was a sister lineage to all other families of the superfamily Gammaroidea. Our study, utilizing all mitochondrial genes with separate partitions, suggests a more complex pattern of Gammaridae evolution, including clades formed by Baikalian Micruropodidae and Crypturopodidae as sister groups to all other families. This deeper structure remains unresolved, even when using whole transcriptome data [49]. The paraphyly of the Gammaridae is confirmed in our study; however, this is not the same as in the study by [50], with Baikalian families as well as the Pontogammaridae. These results support complex and ancient origins of the family Gammaridae, and they are further supported by studies using nuclear data [38,39]. Our study, among others, highlights that the whole superfamily requires taxonomical revision using integrative methods.

Our results suggest that the Eocene could be a backdrop for the burst of diversification observed for the Gammaroidea, resulting in the diversity of families existing today. This early time frame of their speciation is supported by previous studies based on nuclear and mitochondrial sequence data [38]. Interestingly, the diversification of the Pontogammaridae, at least according to our study, takes place between ca. 17 and 8 Ma, and coincides with the time scale of their earliest recorded potential representative, whose fossil record resides in the Upper Sarmatian (ca. 9 Ma: [76,77]).

## 4. Materials and Methods

### 4.1. Material Collection and DNA Isolation

*Dikerogammarus villosus* was collected from its invasive ranges in Poland (50.412 N, 18.108 E, April 2009, *n* = 2), England (52.3024 N, 0.3208 W, September 2016, *n* = 2), and its native range in Turkey (41.316 N, 28.620 E, September 2007, *n* = 1). *Dikerogammarus haemobaphes* was collected in Germany (47.973 N, 11.352 E, May 2011, *n* = 1). *Dikerogammarus bispinosus* was collected in Hungary (47.518 N, 19.042 E, April 2012, *n* = 1). Material was collected using a standard benthic hand-net and identified using available keys (i.e., [78]). The voucher specimens and isolated DNA are stored at the Department of Invertebrate Zoology and Hydrobiology, University of Lodz. Material from England is stored at the Centre for Environment, Fisheries and Aquaculture Science (Cefas) repository. DNA isolation for all specimens followed a standard phenol-chloroform protocol according to a procedure from [79]. To confirm the species identification, the COXI (cytochrome c oxidase subunit 1) fragment was amplified and sequenced, following a procedure from [27]. The COX1 sequences were used in BLAST searches to confirm morphological identification.

### 4.2. Sequencing/Assembly

Isolates with confirmed species identification were tested for DNA quality and quantity using Qubit (Qubit dsDNA BR Assay, ThermoFisher scientific, Waltham, MA USA) and Nanodrop machines. Libraries were prepared from 1 ng of whole genomic DNA using the Nextera XT preparation kit (Illumina) twice with 150 bp paired-end sequencing on an Illumina NextSeq500 sequencer (Illumina, San Diego, CA, USA) in the Biobank Lab, Department of Molecular Biophysics, University of Lodz.

The quality of the reads before and after the pre-processing steps were assessed using FastQC (v0.11.5) [80]. Quality trimming and removal of remaining sequencing adapters was performed with Trimmomatic [81]. Mitobim [82] and NOVOplasty [83] were used to pull out and assemble any mitochondrial genomes from raw DNA reads. The assemblies were then verified through mapping of mitochondrial reads using Bowtie2 [84]. Annotation and visualization were performed using a mitoconstrictor set of tools [85].

In order to provide an extensive set of outgroups within the Gammaroidea superfamily, available databases were also searched, and data downloaded. The mitochondrial genes were obtained from annotated mitochondrial genomes available through NCBI (GenBank). Additionally, unannotated raw transcriptome data (NCBI SRA) were downloaded and assembled anew. A list of species and accession codes of new assemblies and read data are provided in Table S1. The same approach was used to obtain mitogenomic sequences from downloaded SRA data as described above, but the *de novo* assembly was conducted using Trinity [86] and an identification of the mitochondrial sequences was completed with wise2 [87] and infernal [88] using scripts from the mitoconstrictor pipeline. The mitochondrial sequences were then validated through a realignment of the filtered reads. Mitochondrial contigs were further assembled in CLC genomic workbench (QIAGEN, Redwood City, CA, USA) and verified by mapping the raw reads at the final, single mitochondrial contig.

Final annotation was performed using a mitoconstrictor set of tools. The reconstructed mitochondrial COX1 genes (for both: downloaded and own data) and, when available, rRNA genes, were tested with blast vs. GenBank and our own unpublished database to ensure proper species identification of the data.

### 4.3. SNP Recovery

To identify variation between specimens from each of the studied ranges, we performed SNP recovery. All reads per individual were mapped to the reference mitogenome using Bowtie2 [89]. In the case of *D. villosus*, the newly assembled mitogenome from Poland was used as the reference and the reads from native range (Turkey) and invasive range (England) were used for mapping. For *D. haemobaphes*, the already published mitogenome from England (MK644228) was used as a reference and the reads from the newly assembled mitogenome of *D. haemobaphes* from Germany were mapped. For the identification of the SNPs, we used the “Find Variations/SNPs” tool through Geneious 11.1 software [90]. The initial minimum variant frequency was set to 0.35, the *p*-value was calculated using an approximate method, the maximum variant *p*-value was set to 10^−6^ and the minimum strand *p*-value to 10^−5^. The variants were called from both coding and noncoding regions, except for the AT-rich control region.

### 4.4. Phylogenetic Analysis with Molecular Clock Calibration

To analyze the diversification of the Ponto-Caspian gammarids across both a phylogenetic and a temporal context, we reconstructed the amphipod phylogeny using a molecular clock approach. A set of 28 species with available mitogenome data from the superfamily Gammaroidea was used. *Gondogeneia antarctica* (Pontogeneiidae) was added as an outgroup (Table S1). The dataset included 13 protein-coding genes and 2 rRNA genes. The coding genes were aligned via MAFFT [91] with automatic determination of algorithm and gap open penalty set to 2.5, while rRNA were aligned using structural aligner software LocARNA [92]. The alignments were inspected by eye to identify possible misalignments of triplets. In the case of coding genes, they were trimmed to codon positions and, in all cases, trimmed when individual sequences were longer relative to the others, or due to putative miss-annotations (e.g., 12S gene of *G. fossarum*—KY197961). Missing positions at the ends of the sequences were coded with unknown nucleotides to exclude them from the phylogenetic analyses. The whole set of genes reached a length of 12,862 bp.

The phylogeny was reconstructed using Bayesian inference in BEAST 2.6.2 [93]. The evolutionary rates for gammarids, especially for COX1, have been vastly studied and cross-validated using both fossil and geological data (e.g., [94,95,96]), allowing us to apply the general rate of 0.01773 [76] for the COXI gene. Four partitions were used: ND6, having a different substitution model, was treated as a separate partition; 11 protein-coding genes were concatenated in one partition; 12S and 16S rDNA genes with the same substitution model were also treated as one partition, as well as COX1 as a calibrated partition. The substitution model was selected via bModelTest [97]. A birth–death tree model and relaxed clock were set as priors. Four runs of the MCMC, each 50 million generations long and sampled every 5000 generations, were performed and examined for convergence in TRACER 1.7 [98]. All parameters in each run reached the effective sample size (ESS) above 200 and were combined using LogCombiner2.6.2 [93]. The final tree was summarized with TreeAnnotator 2.6.2 [93].

To provide relative rates of evolution for each of the protein-coding genes, the above analysis was also run using all genes as separate partitions. The difference in settings for the clock, in this case a simple “strict clock”, was used for each partition and the length of MCMC was set to 100 million generations.

To provide additional support for the BI topology, we also reconstructed a phylogeny using the maximum likelihood approach through RAxML 8.2.8 [99]. For this analysis, all 15 genes were concatenated. The best-scoring ML tree was produced using the GTR + I + G substitution model. Bipartition information was drawn from phylogeny obtained with the rapid hill-climbing tree search algorithm. Statistical support was estimated with a thorough bootstrap test set to 1000 repetitions. To limit the impact on the phylogeny of highly divergent 3rd codon position, the phylogeny was reconstructed using amino acid sequences. All protein-coding sequences were translated, realigned in MAFFT using BLOSUM65 [91] scoring matrix automatic selection of algorithm, and then concatenated. The tree was reconstructed in RAxML 8.2.8 using the same settings as above.

## 5. Conclusions

We present the first full de novo mitochondrial genomes of *D. villosus* and *D. bispinosus*, as well as a new assembly for *D. haemobaphes* and a partial mitochondrial genome for *P. robustoides*. These mitogenomes serve as a vital resource for the development of new genetic markers for PCR-based identification methods, as well as SNP-based demographic studies. The reconstruction of these mitogenomes shows that Ponto-Caspian gammarids, and their sister lineage *Homoeogammarus*, show a stable structural feature that involves the tRNA-E and tRNA-R being switched in place relative to other gammarids. Aside from this, the pancrustacean structural format is adhered to.

The phylogenetic reconstruction we provide for *Dikerogammarus* spp. and *P. robustoides*, based on the mitogenomes of Ponto-Caspian gammarids and other Gammaroidea, reveals a well-supported group that appears to have originated in the Miocene. Our study supports paraphyly in the Gammaridae family, advocating the need for detailed integrative taxonomic revision of the Gammaroidea superfamily.

## Data Availability

New mitochondrial genomes obtained for Ponto-Caspian amphipods are available in GenBank under accession numbers: *D. villosus*: OK173836-OK173838, *D. haemobaphes*: OK173839, *D. bispinosus*: OK173840, *P. robustoides*: OK173841. Nucleotide sequence of newly obtained assemblies based on GenBank data are available in the Third Party Annotation Section of the DDBJ/ENA/GenBank databases under the accession numbers TPA: BK059223-BK059235 (details in Table S1).

## Supplementary Materials

The following are available online at https://www.mdpi.com/article/10.3390/ijms221910300/s1.
