# Supplementary figures and images for: Mitochondrial Genomes, Phylogenetic Associations, and SNP Recovery for the Key Invasive Ponto-Caspian Amphipods in Europe
Source: Int J Mol Sci. 2021 Sep 24;22(19):10300. doi: 10.3390/ijms221910300 (PMC8509019; doi:10.3390/ijms221910300)

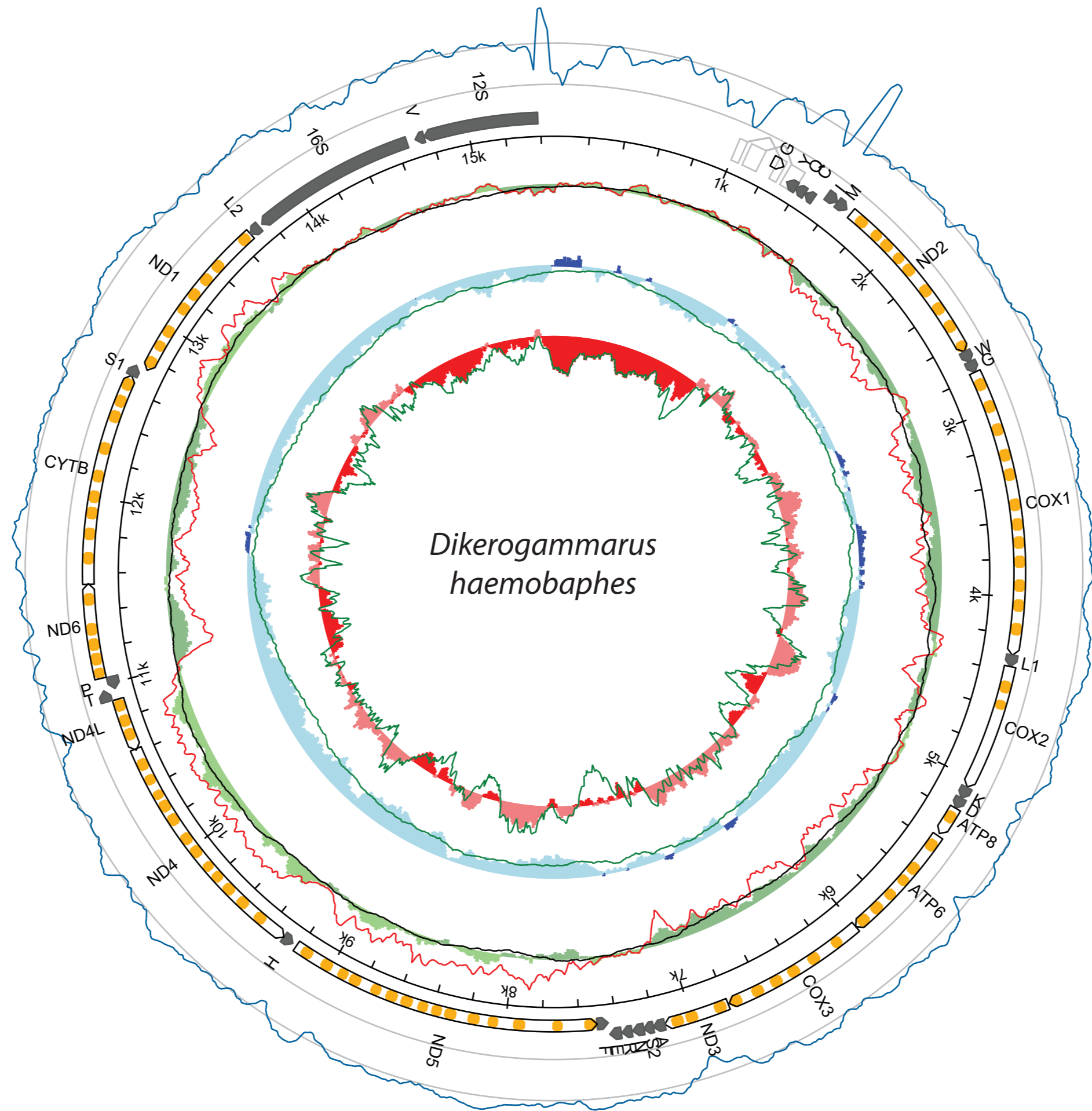

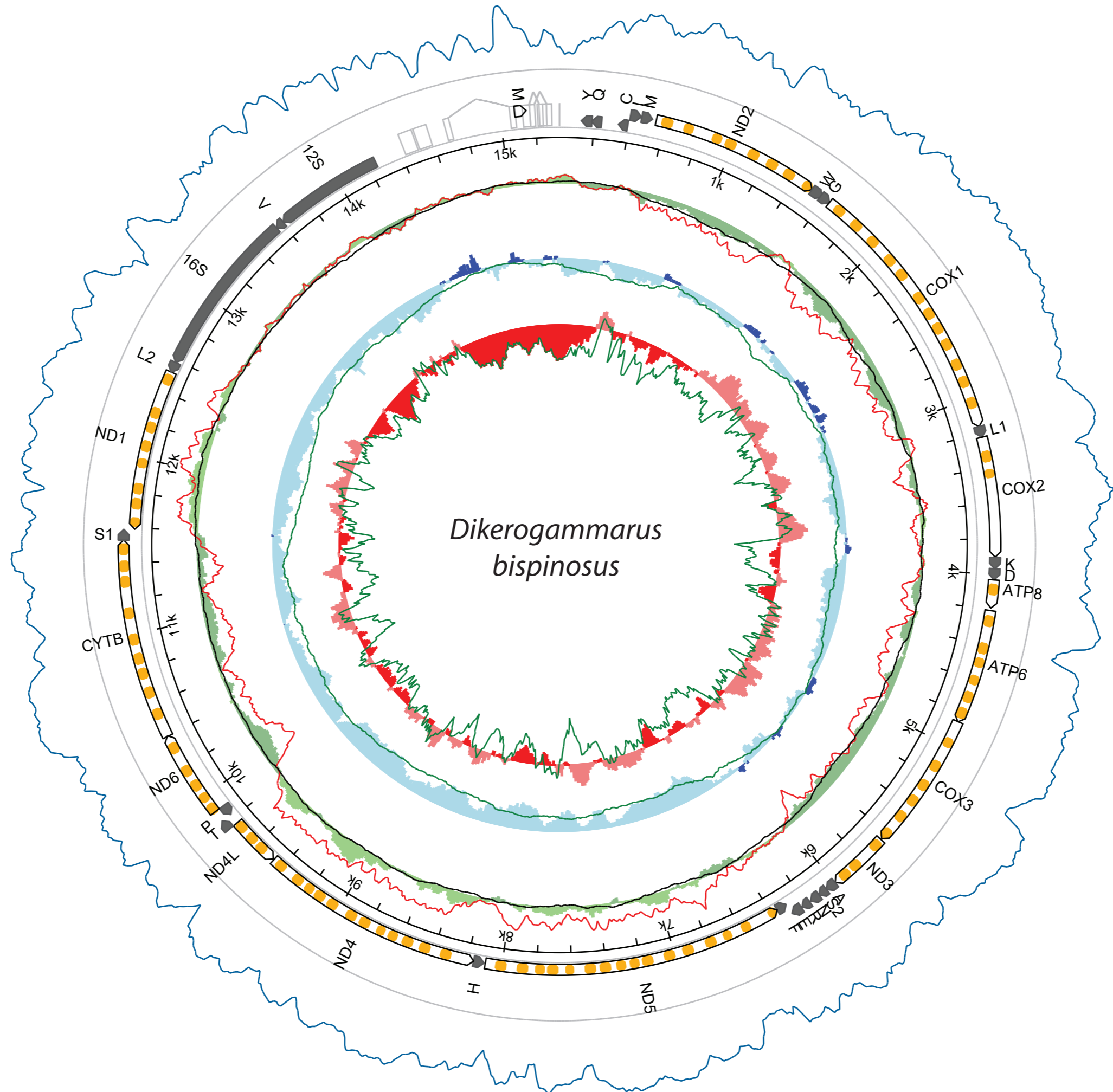

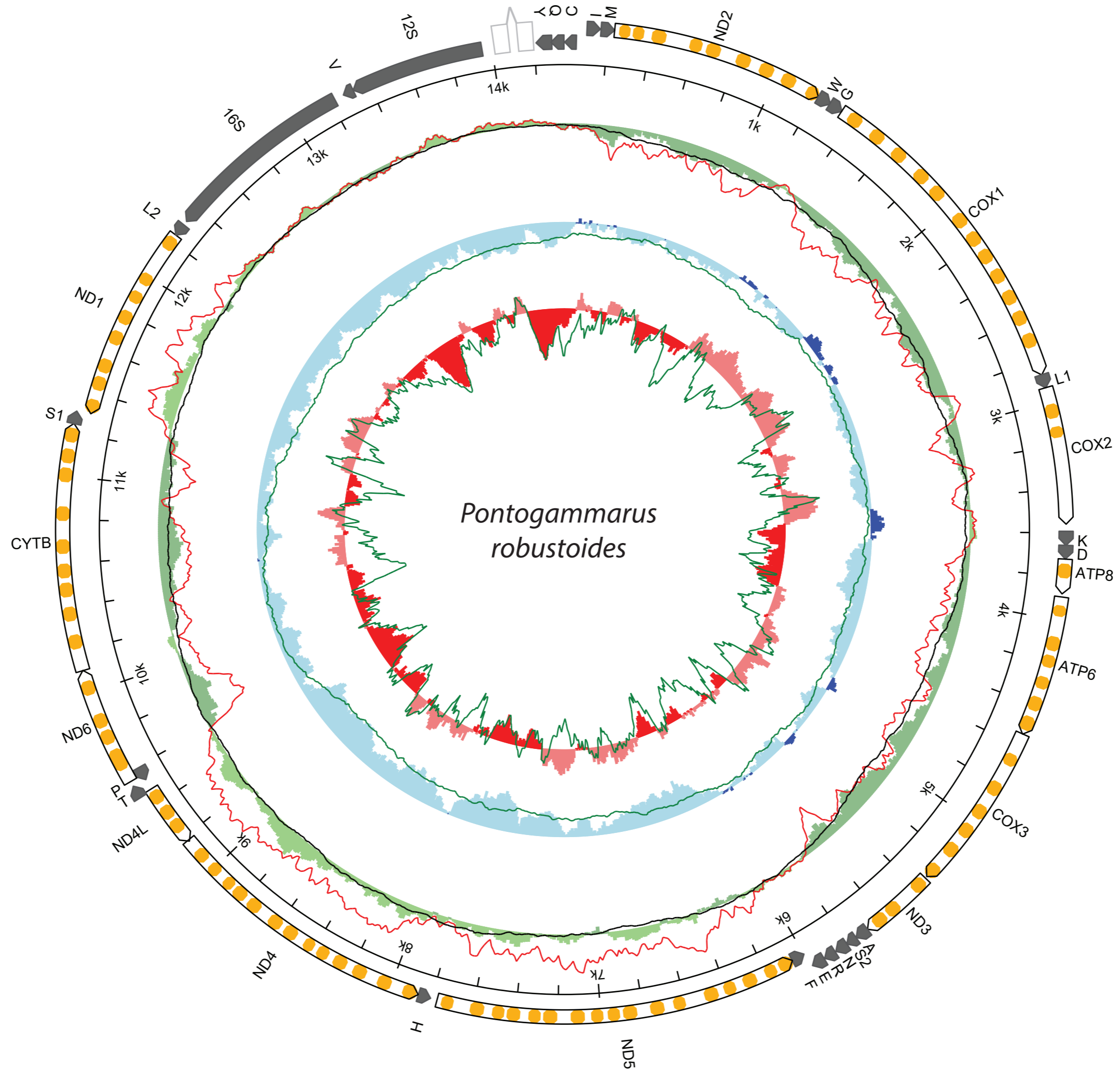

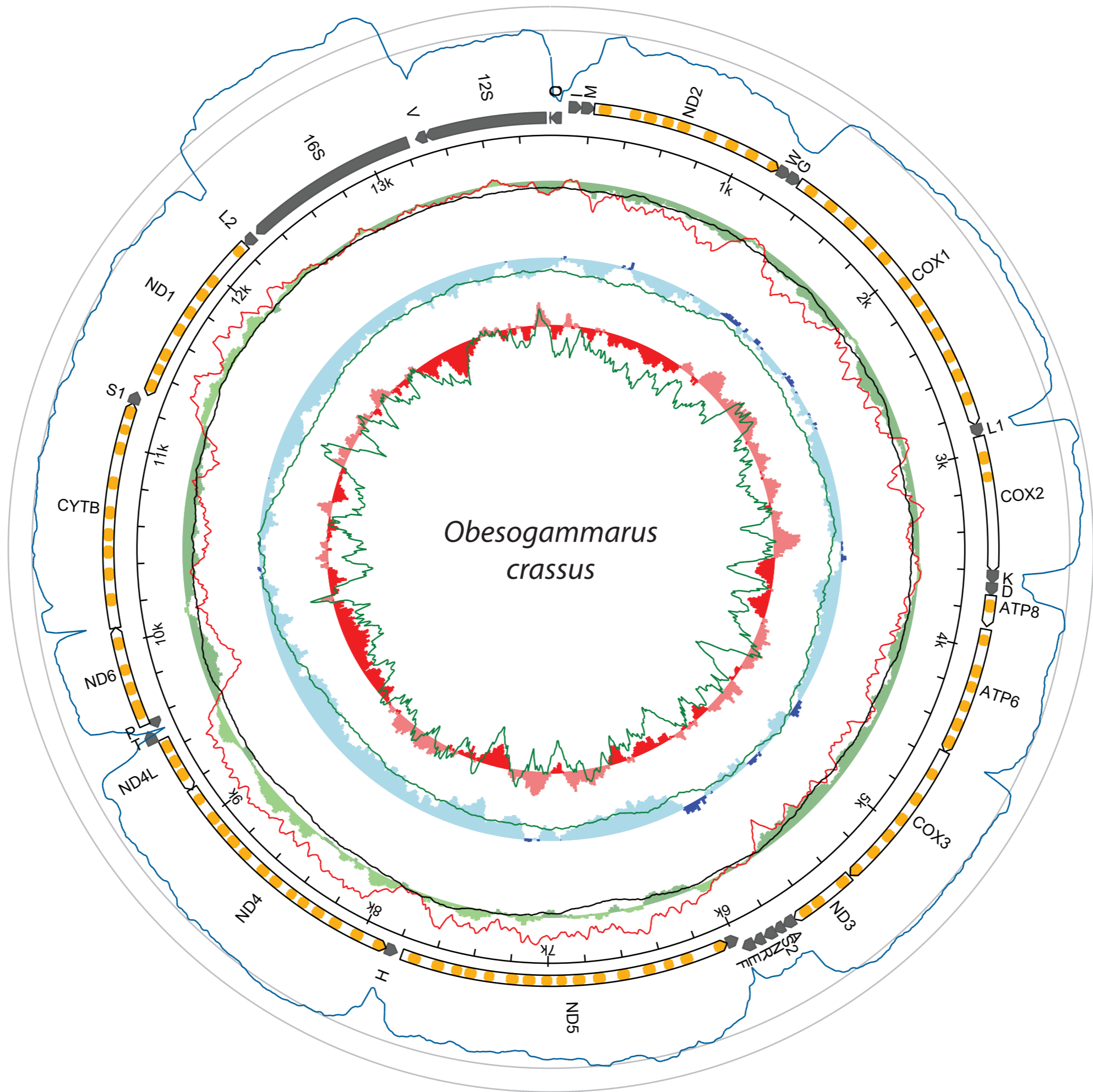

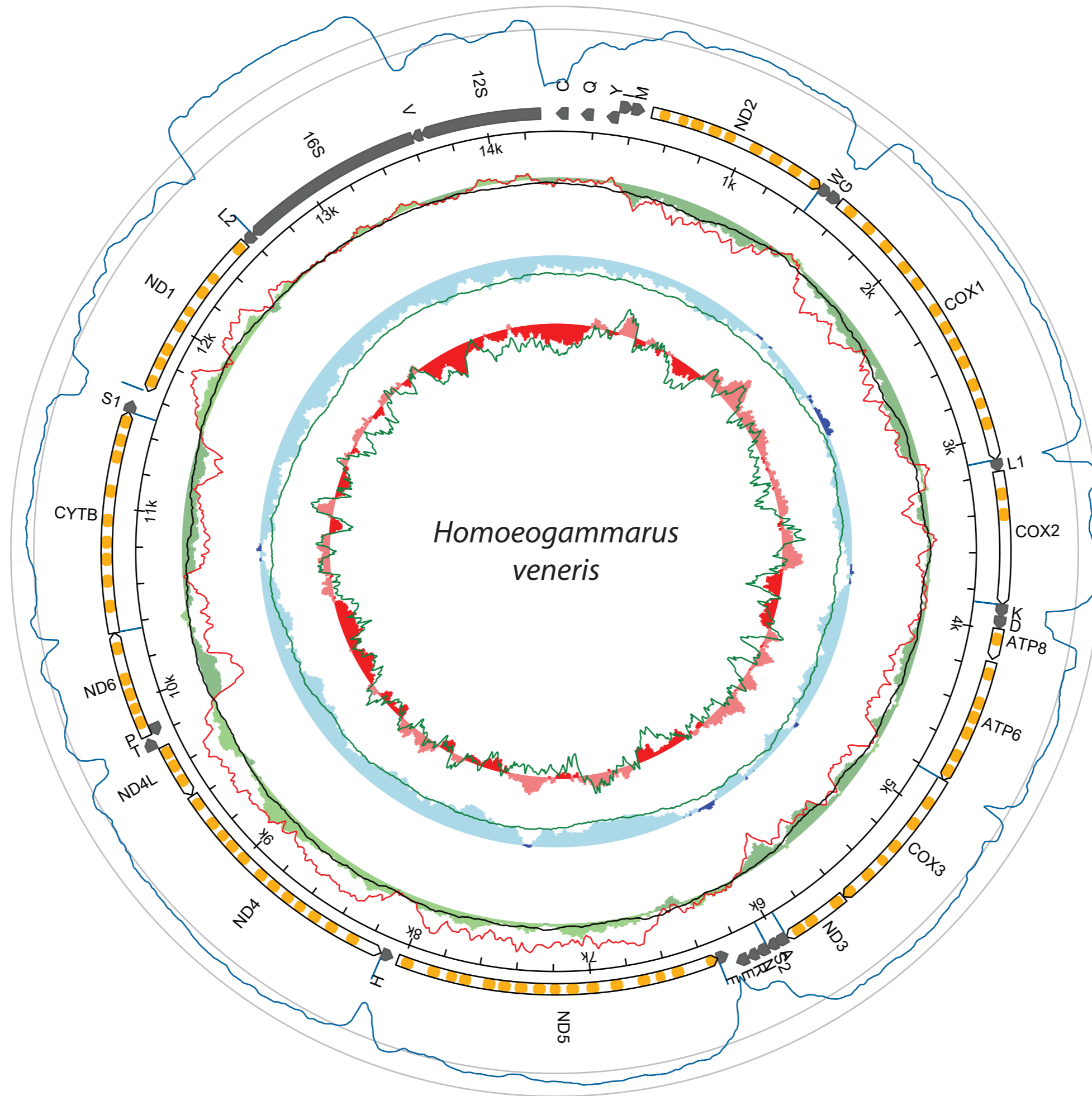

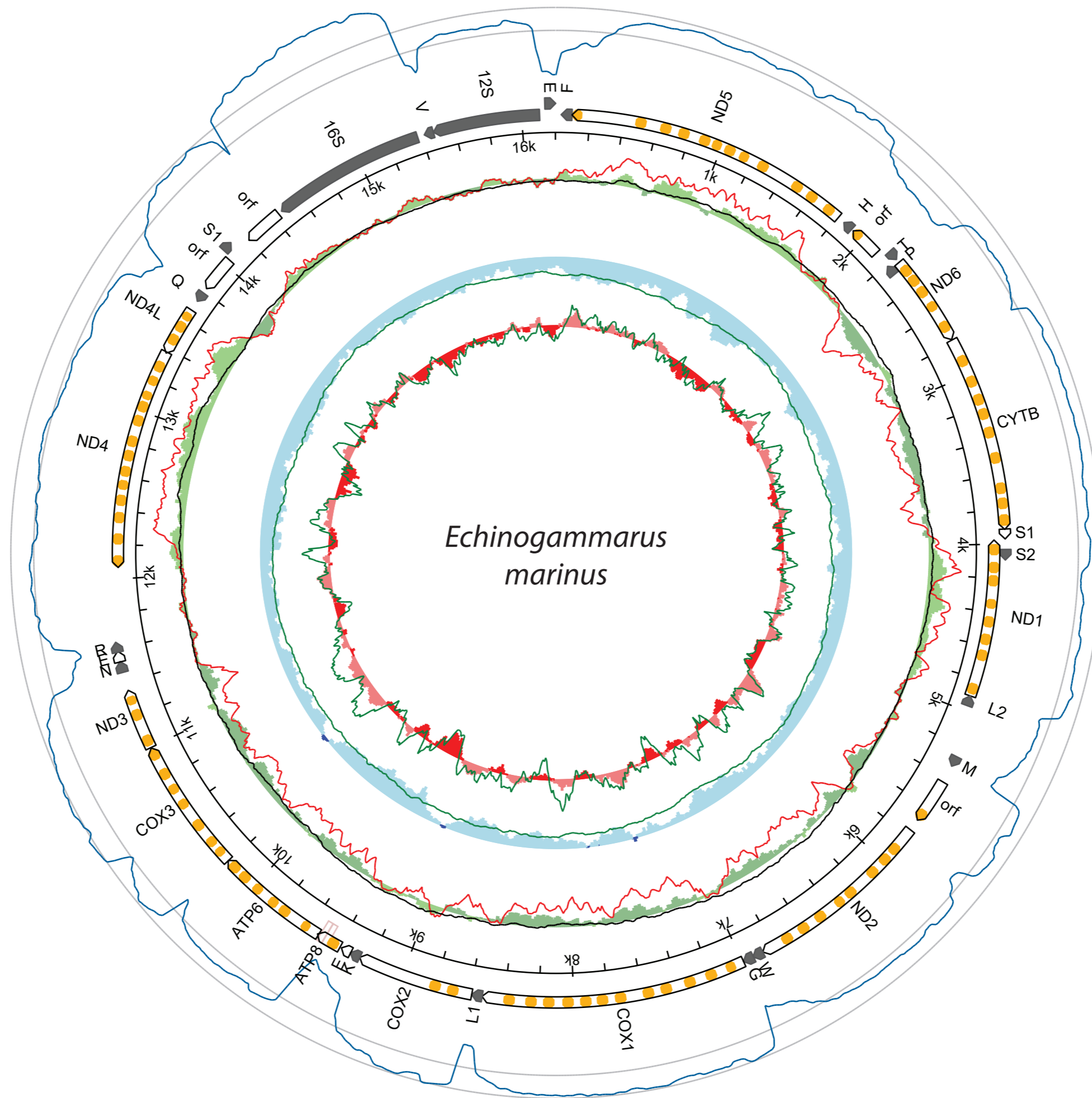

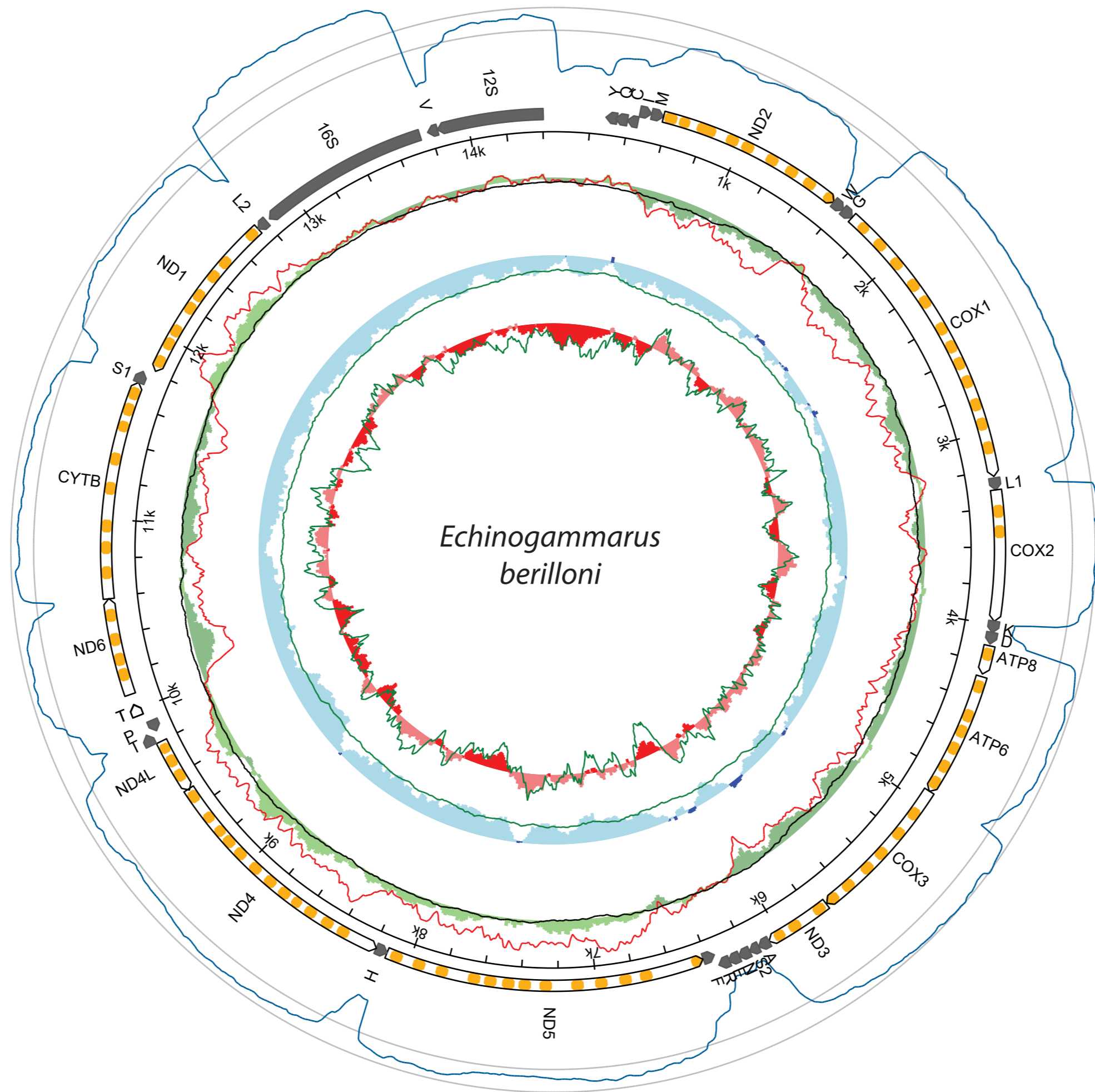

Supplement: Supplementary file 1 [file ijms-22-10300-s001.zip › Fig_S1.pdf]

A

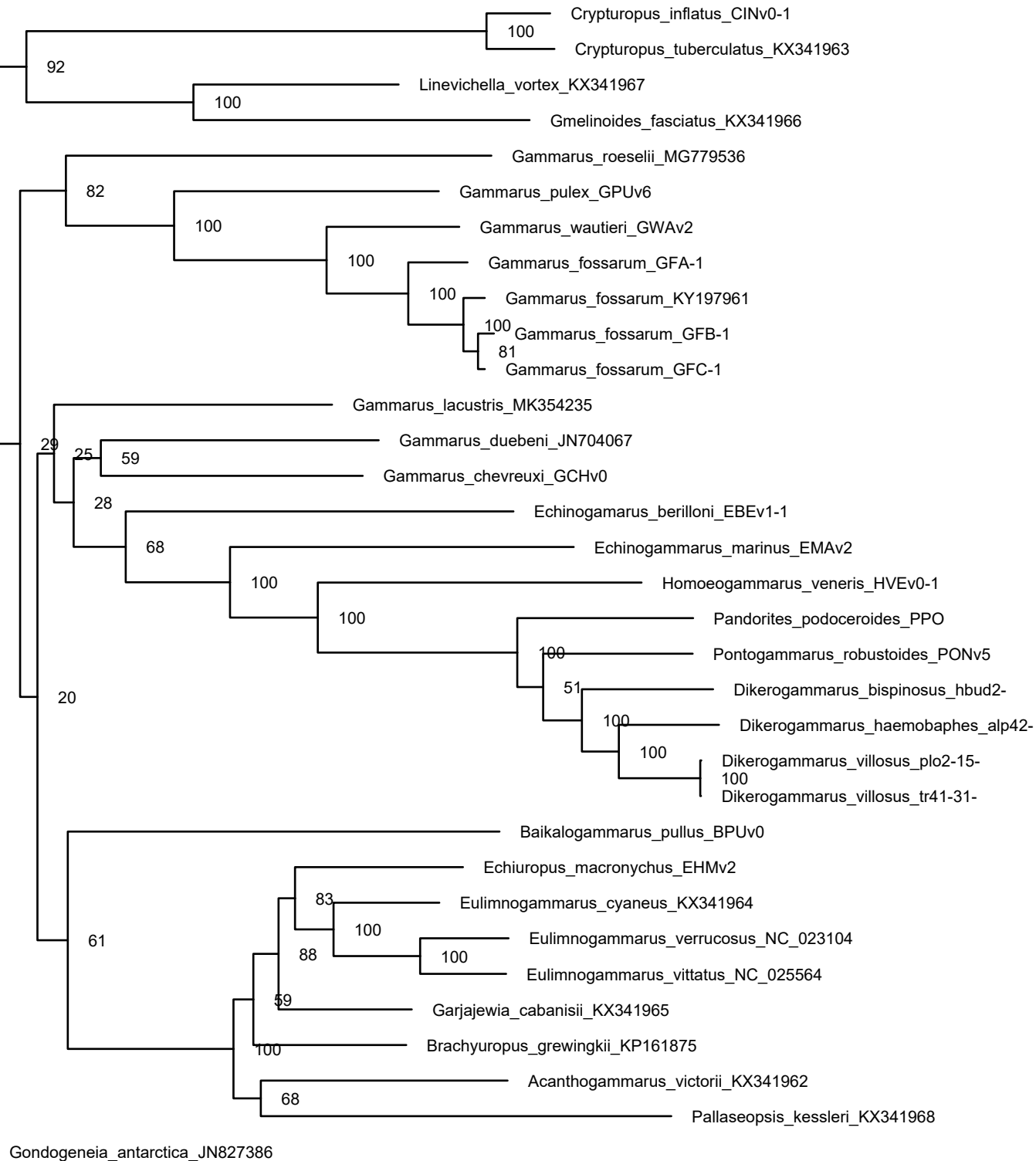

B

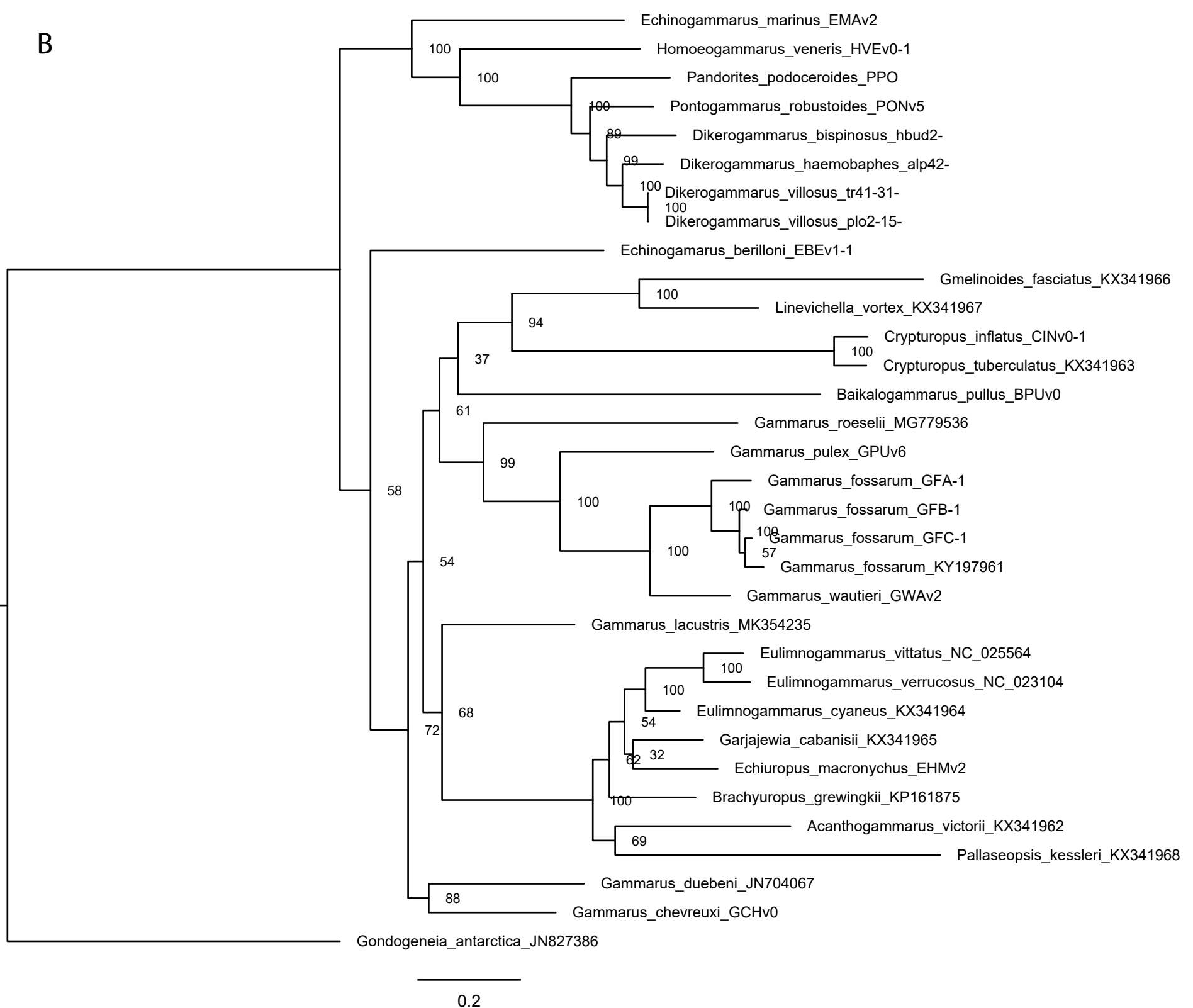

Supplement: Supplementary file 1 [file ijms-22-10300-s001.zip › Fig_S2.pdf]

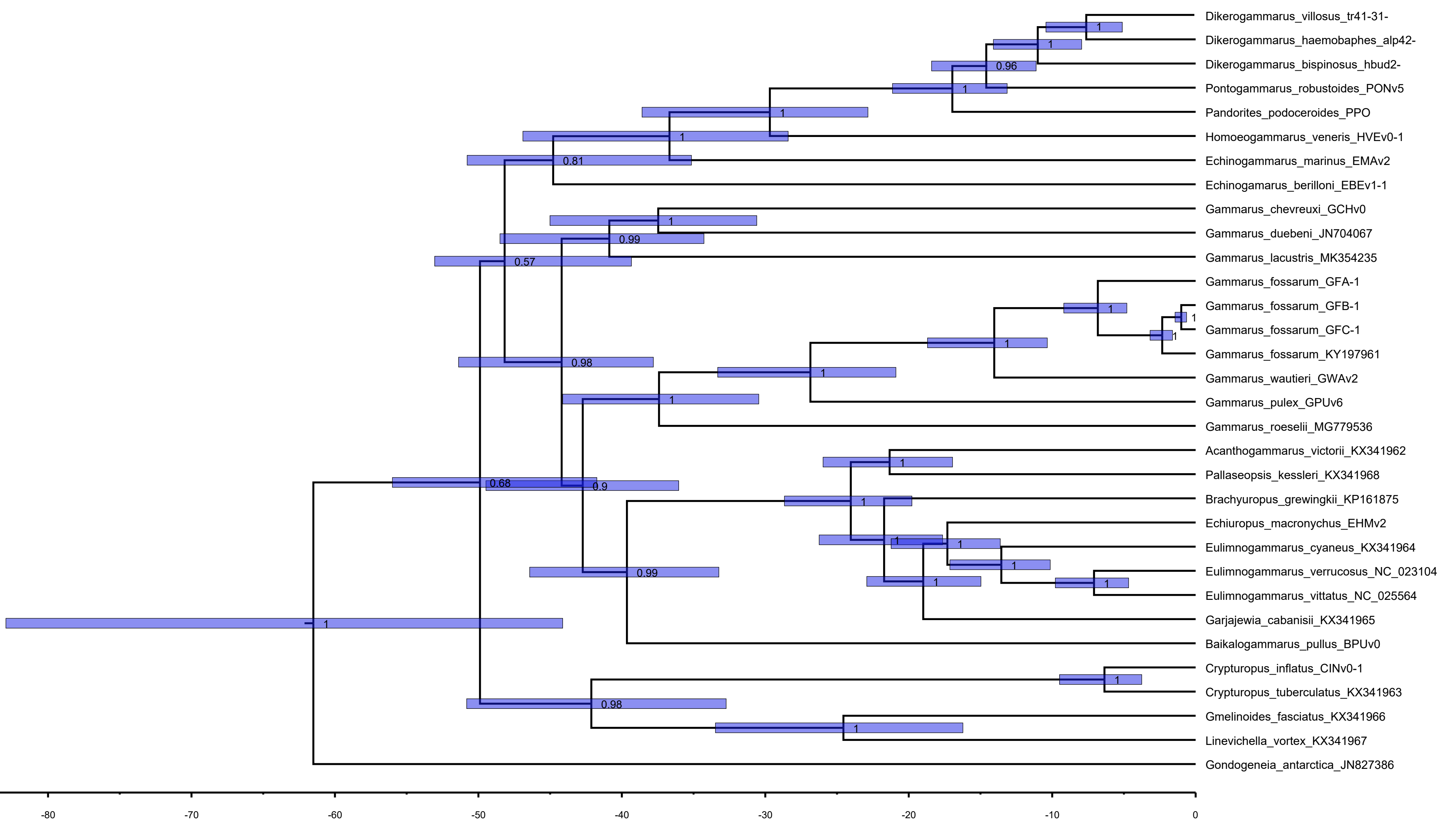

Supplement: Supplementary file 1 [file ijms-22-10300-s001.zip › Fig_S3.pdf]
